# Supplementary material for: Gynodioecy in the common spindle tree (Euonymus europaeus L.) involves differences in the asymmetry of corolla shapes between sexually differentiated flowers
Source: PeerJ. 2020 Feb 14;8:e8571. doi: 10.7717/peerj.8571 (PMC7025705; doi:10.7717/peerj.8571)
Supplement: Figure S1 — (A) A single corolla configuration and all four transformed copies produced by reflection across two axes of symmetry. (B) Three types of principal components yielded by the principal component analysis of the Procrustes aligned quadrupled dataset consisting of transformed (i.e. reflected and re-labelled) copies of individual configurations. [file peerj-08-8571-s002.pdf]

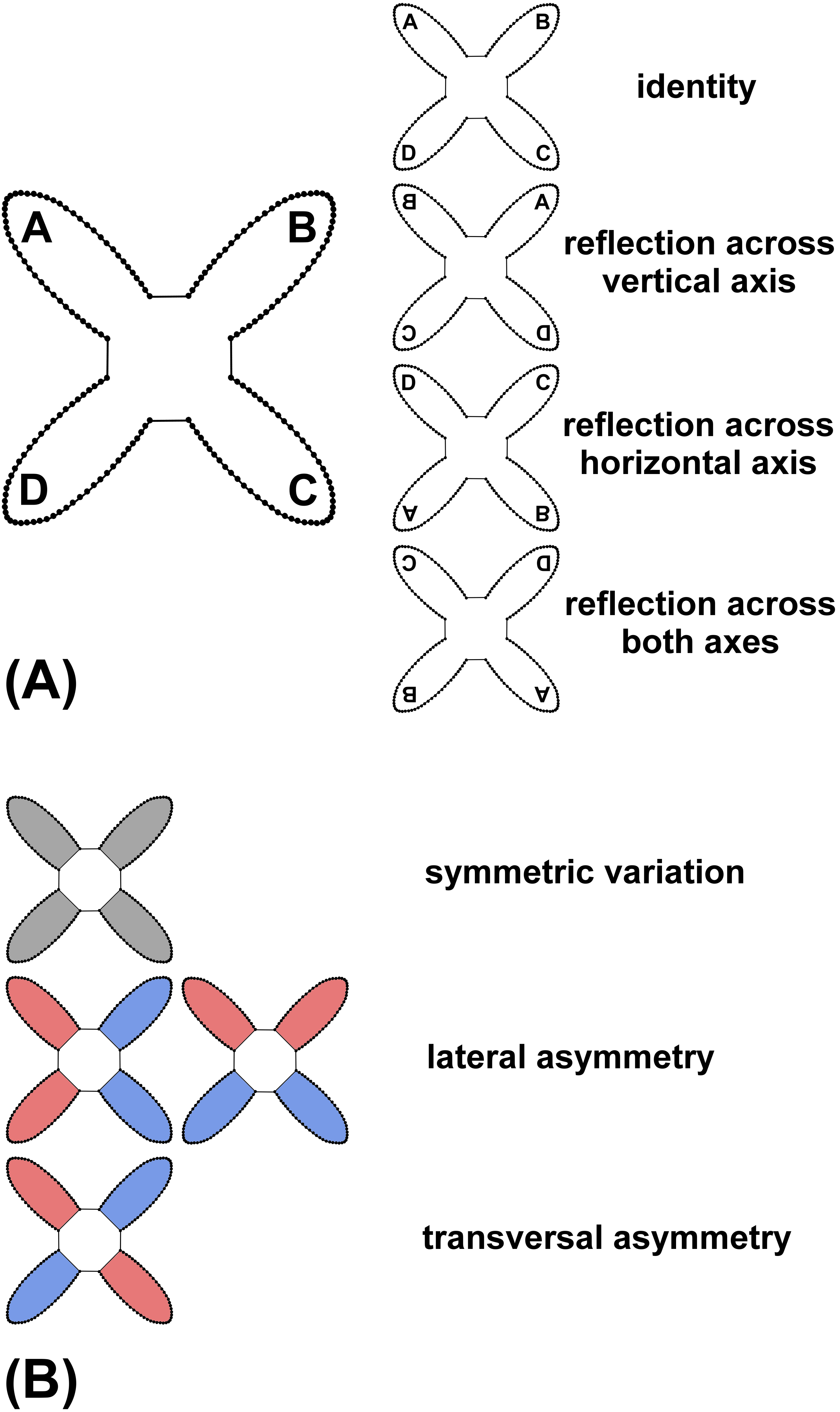
A
B
D
C
identity
reflection across vertical axis
reflection across horizontal axis
reflection across both axes
(A)
symmetric variation
lateral asymmetry
transversal asymmetry
(B)
